# Heterogeneous protein dynamics links to mitochondrial activity, glucose transporter, and ALDH cancer stem cell properties

**DOI:** 10.1186/s12885-025-14460-x

**Published:** 2025-07-01

**Authors:** Martin Krkoška, Zuzana Tylichová, Pavlína Zatloukalová, Petr Müller, Bořivoj Vojtěšek, Philip John Coates

**Affiliations:** Research Centre for Applied Molecular Oncology, Masaryk Memorial Cancer Institute, Žlutý kopec 7, Brno, 656 53 Czech Republic

**Keywords:** Squamous cell carcinoma, Cancer stem cells, Proteosynthesis, Protein degradation, Mitochondrial membrane potential, Glucose transporter

## Abstract

**Background:**

Cancer stem-like cells (CSCs) represent a subset of tumor cells that have the ability to self-renew, a long lifespan and a relatively quiescent phenotype, and show resistance to conventional therapies. Various markers are used to identify CSCs, and have shown that different CSC subtypes may be present within a tumor. One functional property of CSCs is their relative lack of proteasomal activity compared to the tumor bulk.

**Methods:**

We introduced an unstable fluorescent molecule into FaDu oropharyngeal squamous cell carcinoma cells and analyzed the association of proteasome activity with aldehydehyde dehydrogenase (ALDH) activity as another common CSC marker, and with other stem-cell related properties of glucose metabolism. We also analyzed publicly available gene expression profiling data of ALDH^+^ CSCs for alterations in mRNAs associated with proteostasis.

**Results:**

We show that FaDu CSCs identified by low proteasome activity are associated with the population identified by high ALDH activity. Futher characterization shows that these CSCs have a relatively high mitochondrial membrane potential and low levels of glucose transporter, indicating a non-Warburg metabolic phenotype. We also show that proteasome-low FaDu CSCs exhibit decreased rates of protein synthesis. Gene expression profiling of other cancer cell lines reveal common statistically significant differences in proteostasis in ALDH^+^ CSCs compared to the bulk of the tumor cells, including reduced levels of Hsp70 and/or Hsp90 in CSCs defined by ALDH, together with reduced levels of *UCHL5* mRNA.

**Conclusions:**

These data provide additional insights into the functional characteristics of proteasome-low/ALDH-high CSCs, indicating a metabolic phenotype of reduced reliance on aerobic glycolysis and a decreased protein synthesis rate. We also identify specific chaperone and ubiquitin ligase activities that can be used to identify CSCs, with corresponding implications for therapeutic strategies that target CSCs through their altered metabolic properties.

**Supplementary Information:**

The online version contains supplementary material available at 10.1186/s12885-025-14460-x.

## Background

Cancer stem-like cells (CSCs) are considered as the unique cell population that is required to maintain overall tumor growth and to act as metastasis-initiating cells. CSCs are resistant to common anticancer therapies due to their slow proliferation, active export of cytotoxic chemotherapeutic agents, and increased DNA repair capacity. Like normal somatic stem cells, CSCs represent only a fraction of tumor cells [4, 12, 27, 51] and are identified mainly by markers such as cell surface proteins (CD44^+^/CD24^−^, CD133^+^, or integrins), high aldehyde dehydrogenase (ALDH) activity, the ability to form spheres in suspension cultures, or the ability to efflux dyes to produce a side population [45, 48]. However, these approaches identify different tumor cell subpopulations, indicating that CSCs are heterogeneous and that their properties vary depending not only on cancer type but also within different regions of the same tumor [27, 38, 47, 48]. Thus, it is necessary to target all CSC subpopulations within a tumor to prevent growth and recurrence after treatment [82], requiring in-depth characterization of CSC subsets to identify effective treatment options. It is also known that CSCs are able to transition from one subtype to another depending in part on their microenvironmental conditions (reviewed in [4, 59, 66], and that both normal stem cells and CSCs exist in a hierarchy of dormant “reserve” cells and more proliferative “repopulating” stem cells [24, 64, 77]. Consequently, adult stem cells and CSCs cannot be universally identified by the expression of individual stem cell-related markers, and it is crucial to identify general functional properties of these cells [13, 87].

One proposed feature of CSCs is their relatively low level of proteasome activity compared to the main population of cells that comprise the tumor bulk [83], and low proteasome activity cells of different tumor origins exhibit CSC-like properties, including treatment resistance [15, 33, 57, 60]. Importantly, the presence of low proteasome activity cells is a predictor of patient prognosis, and specific targeting of this cell population leads to tumor regression in vivo [40, 43, 84]. These data indicate that CSCs show differences in proteostasis, and their lack of proteasome activity may reflect a general reduction of protein turnover rates associated with their relatively quiescent state compared to most cells in the tumor mass. Indeed, protein synthesis rates are low in normal hematopoietic stem cells, and have been suggested as a defining and necessary feature of these cells [10]. Furthermore, there is evidence in other tissues that both normal stem cells and CSCs show reduced protein synthesis rates, and that a global reduction of protein synthesis and altered translational programmes promote stem cell functions and tumorigenesis [5, 34]. These considerations may underlie the observations that whereas most tumor cells are addicted to abnormally high proteasome activity [78], low proteasome activity identifies CSCs [57, 83]. In addition, it has been suggested that stem cell properties are characterized by low global translation rates despite high levels of ribosome biogenesis [36, 67], and high ribosome biosynthetic rates are associated with CSCs in colorectal cancer [55]. Thus, normal adult stem cells and CSCs may show reduced protein synthesis and reduced protein degradation rates, or these may be uncoupled aspects of proteostasis in neoplastic cells.

Another characteristic feature of tumors is the metabolic reprogramming of tumor cells, which is required for the anabolic production of intermediates to supply macromolecule synthesis demands to maintain rapid cellular growth. Part of this reprogramming involves aerobic glycolysis, also known as the Warburg effect [20, 30, 32, 61]. In turn, Warburg metabolism in cancer requires higher levels of glucose uptake than normal cells for energy production, forming the basis of ^18^F-FDG PET imaging for cancer detection. Whether CSCs with their comparatively decreased biosynthetic demands also show high glucose transport and increased aerobic glycolysis is unclear.

Here, we investigated proteasomal activity coupled with the levels of CSC markers and other metabolic characteristics in invidual cells of a clonal population of head and neck squamous cell carcinoma (HNSCC) cells. Our data indicate that the subpopulation of tumor cells with low proteasomal activity shows high ALDH activity, compatible with their CSC phenotype. Low proteasome activity also correlates with high mitochondrial membrane potential and low glucose transporter levels, indicating a non-Warburg metabolic profile in this CSC population. Finally, these cells show reduced protein synthesis, defining an altered regulation of proteostasis in this CSC population. These findings were extended by analysis of publicly available gene expression profiles of ovarian tumor cell lines, revealing a consistent association of ALDH^+^ CSCs with altered protein chaperones and protein degradation pathways compared to the bulk of the tumor cells.

## Results

### Development of a system to assess proteasomal function

**Fig. 1:**
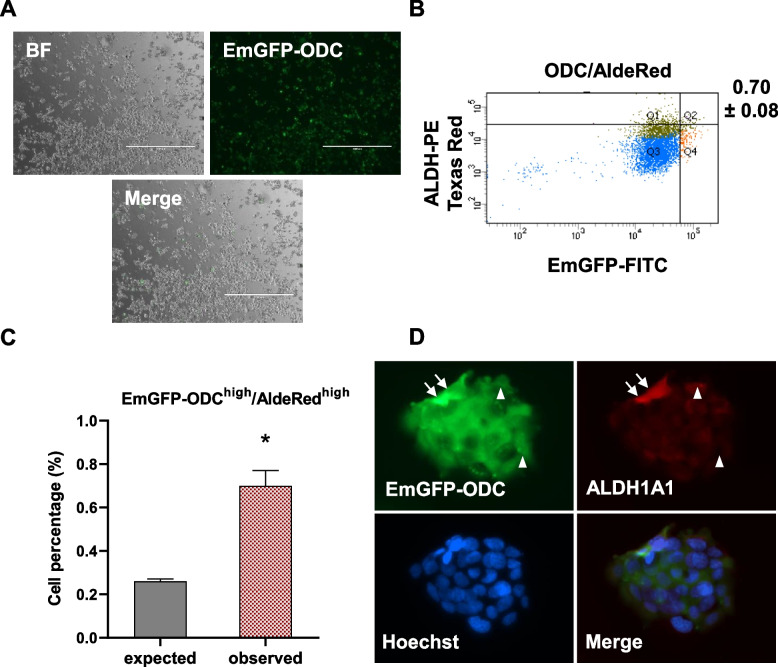
Low proteasome activity associates with high ALDH activity. **A** bright field (BF) and fluorescent images of FaDu cells transfected with the PB-EF1a-N-EmGFP-ODC1(418–461)-PURO-GWs plasmid. Clones were subsequently selected via continuous puromycin treatment (2 μg/ml), single cell sorted and expanded. Scale bar: 1000 um. **B** Flow cytometry of FaDu EmGFP-ODC cells stained for ALDH enzyme activity using the AldeRed Detection Kit. Cell populations are color-coded as follows: cells negative/low for both ALDH and EmGFP (blue), ALDH-positive cells (olive), and EmGFP-ODC-high (low proteasome activity) cells (red). **C** The percentage of double positive cells observed experimentally (upper right) compared to the expected value if the two parameters are unrelated (mean ± SEM, *n* = 3 biological replicates) * *p* < 0.05. Please refer to the Methods section and supplementary information for a detailed explanation of the gating procedure and the terms “expected” and “observed” values. **D** FaDu EmGFP-ODC cells (green) stained for ALDH1A1 (ab195255, red). Nuclei are stained with Hoechst 33,342 (blue). Cells marked with arrows indicate co-localization, while arrowheads represent cells that are negative/weak for both markers. Representative images from at least three independent experiments

Initially, we engineered a FaDu HNSCC cell system for evaluating proteasomal activity. FaDu cells were transfected with PB-EF1a-N-EmGFP-ODC1(418–461)-PURO-GWs plasmid (Fig. S1) that encodes EmGFP fused to the murine ODC degron sequence at the C-terminus, which targets EmGFP for rapid degradation in the proteasome. Previous studies have shown that CSCs show relatively low proteasome activity, leading to high levels of fluorescence [57, 83]. Transfected cells were selected with puromycin (2 μg/ml) for 7 days before single-cell sorting and expansion and testing for EmGFP positivity (Fig. 1A). Short-term treatment with the proteasome inhibitor MG-132 (5 uM, 4 h) increased fluorescence levels, providing functional validation for dependency on proteasomal activity (Fig. S2). This system was employed to investigate the relationships beteween reduced proteasomal activity, ALDH activity, mitochondrial activity, glucose transporter levels and protein synthesis rates.

### Low proteasome activity shows a positive association with ALDH activity

ALDH activity is a commonly used marker of normal stem cells and CSCs, including HNSCC models such as FaDu cells [8, 25, 39, 73]. Therefore, we investigated whether EmGFP levels as an indicator of proteasome activity are associated with ALDH activity in individual cells using flow cytometry (Fig. 1, S2-3). As a control for ALDH assay specificity, there was a decrease in the number of ALDH^+^ cells in the presence of the inhibitor DEAB (Fig. S3). Based on previous observations that CSCs represent a variable proportion of the tumour cell population (generally estimated as approximately 2% to 10% but ranging from less than 0.1% to as much as 40%, depending on the assay system and cancer type [17, 25, 31, 81], we used a 5% cut-off to enrich for the CSC population as described in Liu Y et al. [48]). Thus, we asked whether the EmGFP^high^ population of cells (low proteasome activity) were also positive for ALDH activity using the highest 5% for each marker as the population containing the putative CSCs. In our studies, the cells are not FACS-purified for subsequent analysis but remain as a mixture of all cells, with analysis performed on each individual cell in the mixture to ensure that all cells are under identical microenvironmental conditions at the time of measurement. From our gating procedure, the population of cells in the double-gated region will contain 0.25% of the total cells (5% of 5%) if the two paremeters are not related to each other (the null hypothesis). This is denoted as the “expected” population of cells in the figure graphs. The “observed” cell percentage then represents the experimentally determined percentage of cells within the double-gated population, which is statistically compared to the expected value using data from the three biological replicates to test the probability that the null hypothesis is valid. Please see Materials and methods for more details on the statistical approach, including how the expected values are determined using manual gating at approximately the 5% level, producing a mean and error value for the expected values. Using these criteria, the experimentally observed EmGFP-ODC^high^ population contained a higher than expected population of ALDH^high^ cells predicted by the null hypothesis, indicating that the EmGFP-ODC^high^ cell population was enriched for ALDH^high^ cells (*p* < 0.05) (Fig. 1B-D). We also used immunoflourescence to visualize ALDH1A1 (a CSC-related isoenzyme, [79]) within EmGFP-ODC cells, again showing a positive correlation of co-fluorescence for EmGFP and ALDH1A1 protein (Fig. 1D). These results indicate that CSCs identified by low proteasome activity exhibit high ALDH levels and activity.

### Cells with low proteasome activity show high mitochondrial activity

**Fig. 2:**
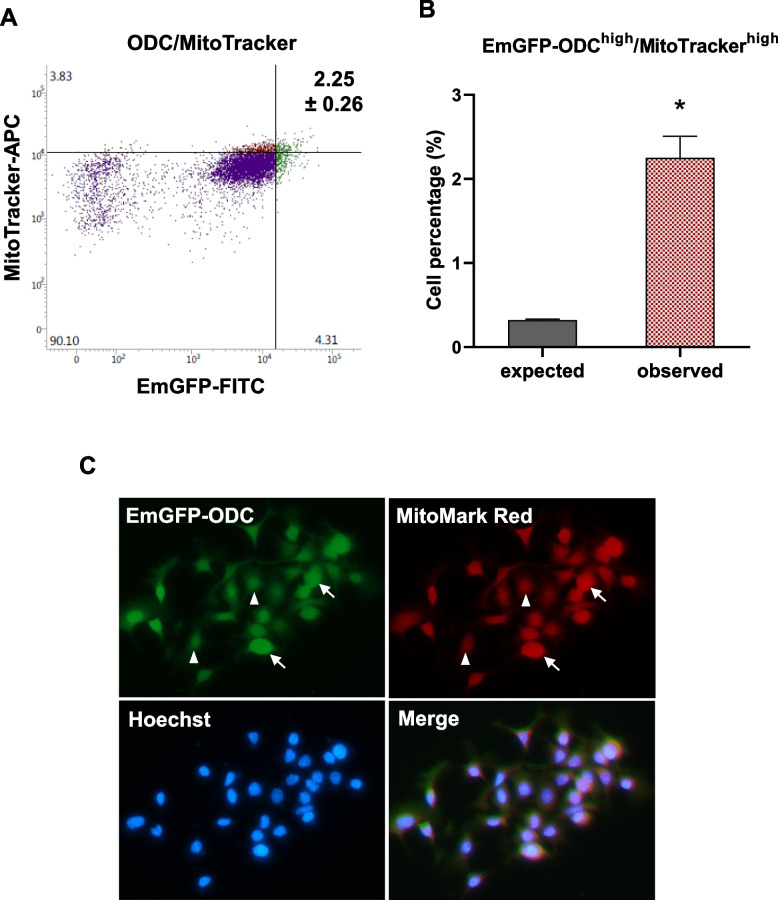
Cells with low proteasome activity also show high mitochondrial activity. **A** FaDu EmGFP-ODC cell counts (determined by flow cytometry) assessed for mitochondrial activity (MitoTracker Deep Red FM). Cell populations are color-coded as follows: cells negative/low for both MitoTracker and EmGFP (purple), MitoTracker-high cells (red), and EmGFP-ODC-high (low proteasome activity) cells (green). **B** The percentage of double-positive cells observed (upper right) compared to the expected values if there is no association (mean ± SEM, *n* = 3 biological replicates) * *p* < 0.05. Please refer to the Methods section and supplementary information for a detailed explanation of the gating procedure and the terms “expected” and “observed” values. **C** Mitochondrial membrane potential of FaDu EmGFP-ODC cells (green) stained with MitoMark Red (red) and Hoechst 33,342 (blue). Cells marked with arrows indicate co-localization, and arrowheads represent cells that are negative/weak for both markers. Representative images from at least three independent experiments

Increased mitochondrial membrane potential (MMP) and activity have previously been reported to determine stem-like phenotypes [74]. The relationships between MMP and proteasomal activity were investigated using MitoTracker Deep Red for flow cytometry and MitoMark Red for microscopy, each of which accumulate in cells dependent on MMP [63]. The population of EmGFP-ODC^high^ cells were also MitoTracker Deep Red^high^ (Fig. 2A-B). Co-localization of EmGFP fluorescence with MitoMark Red fluorescence using microscopy was consistent with these data (Fig. 2C).

### Cells with low proteasome activity show low glucose transporter levels

**Fig. 3:**
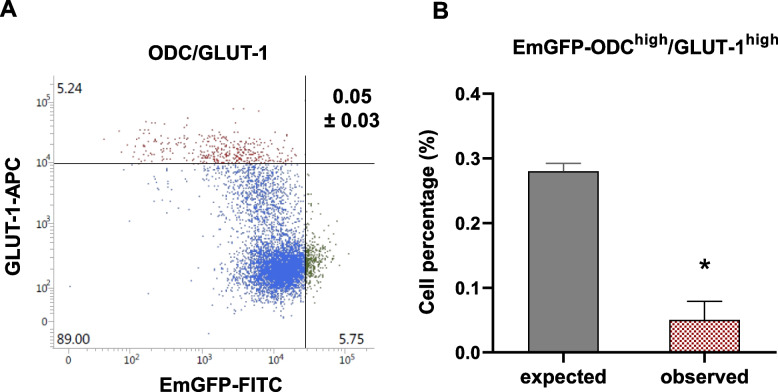
Low glucose uptake cells have low proteasome activity. **A** Flow cytometry of FaDu EmGFP-ODC cells stained for GLUT-1 (Alexa Fluor® 647-conjugated antibody). Cell populations are color-coded as follows: cells negative/low for both GLUT-1 and EmGFP (blue), GLUT-1-high cells (red), and EmGFP-ODC-high (low proteasome activity) cells (green). **B** The percentage of double-positive cells (upper right) compared to the expected values (mean ± SEM, *n* = 3 biological replicates) * *p* < 0.05. Please refer to the Methods section and supplementary information for a detailed explanation of the gating procedure and the terms “expected” and “observed” values

We also investigated the relationship between EmGFP and glucose transport capacity, represented by the GLUT-1 glucose transporter, the major active transporter of glucose in cancer [75]. The average levels of GLUT-1 varied by approximately two orders of magnitude in individual cells, with a distinct GLUT-1^high^ sub-population (Fig. 3A). GLUT-1^high^ cells were depleted from the EmGFP^high^ cell population (Fig. 3B), indicating that CSCs identified by low proteasomal activity have lower glucose transport capacity than the majority of tumor cells.

### Low proteasome activity is associated with decreased protein synthesis rate

**Fig. 4:**
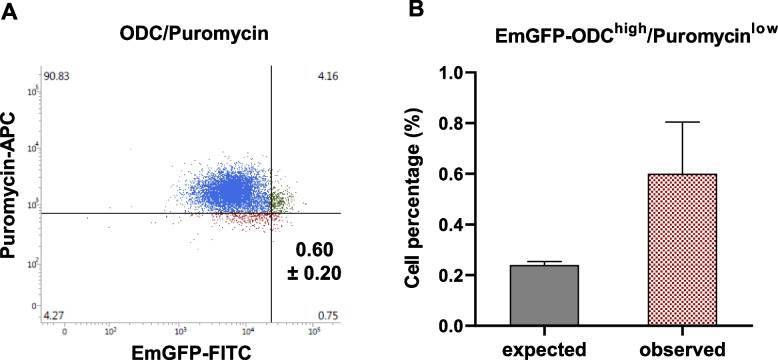
Correlation of proteasome activity with protein synthesis. **A** FaDu EmGFP-ODC cells stained for puromycin incorporation (2 μg/ml, 15 min). Cell populations are color-coded as follows: cells that are classified as not puromycin-low or EmGFP-high (blue), puromycin-low cells (red), and EmGFP-high (low proteasome activity) cells (green). The bottom right quadrant indicates cells with low proteasome activity and low protein synthesis rate. **B** The percentage of cells with low proteasome activity and low protein synthesis rate compared to the expected values (mean ± SEM, *n* = 3 biological replicates). Please refer to the Methods section and supplementary information for a detailed explanation of the gating procedure and the terms “expected” and “observed” values

Previous studies have indicated that normal stem cells in the haematopoietic system and squamous skin epithelium show reduced protein synthesis rates [5, 10, 67]. Therefore, we assessed the correlation between low proteasome activity and protein synthesis rate measured by the incorporation of puromycin into newly synthesized proteins (Fig. 4, S5). The high proteasome activity CSC population of EmGFP-ODC^high^ cells showed a 2.5-fold enrichment of cells with low protein synthesis rates (puromycin^low^ cells), although this did not reach statistical significance (Fig. 4).

### *ALDH*^+^*CSCs commonly show altered protein chaperone and protein degradation compared to the tumor bulk*

**Fig. 5:**
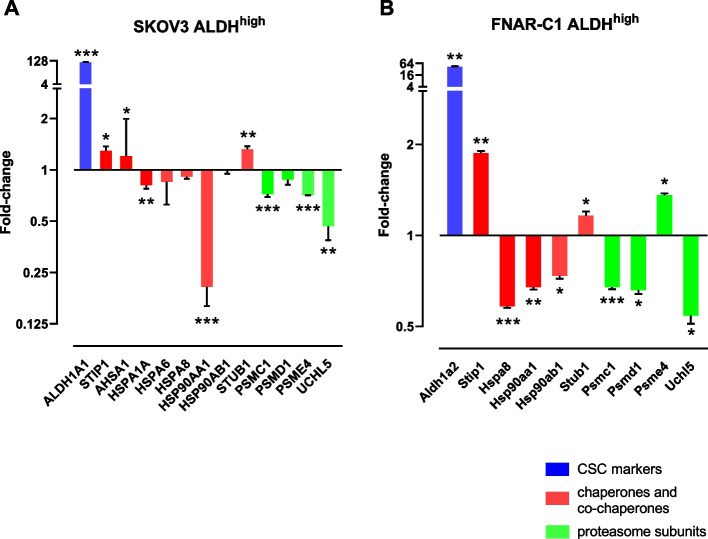
Correlation of proteostasis with ALDH activity in ovarian cancer cells. Gene expression profiling data of the indicated mRNAs in ALDH^high^ compared to ALDH^low^ cells. **A** SKOV-3 high grade human ovarian cystadenocarcinoma cells. **B** FNAR-C1 rat endometrioid ovarian carcinoma cells. Values on the y axis represent the fold-hange in mRNA levels in ALDH^low^ compared to ALDH.^high^ cells plotted on a log(2) scale (mean ± SEM, *n* = 3 biological replicates). * *p* < 0.05; ** *p* < 0.01; *** *p* < 0.001

To extend our experimental observations of proteasome^low^ cells with ALDH^+^ CSCs in the FaDu squamous cancer cell line, we examined previous data of two ovarian cancer cell lines in which the ALDH^+^ population had been isolated and gene expression profiling performed. We retrieved data for mRNAs that code for major chaperones and co-chaperones involved in protein folding and stabilization, along with subunits of the proteasome and ubiquitin ligase regulation. These data revealed consistent small but statistically significant alterations in chaperones in the ALDH^+^ CSC population, with decreased levels of the major Hsp90 form, Hsp90A (encoded by *HSP90AA1*). Analysis of the major stimulatory and inhibitory Hsp90 co-chaperones showed increased levels of both the pro-folding chaperone, *STIP1*, and the co-chaperone ubiquitin ligase *STUB1* (also called CHIP). We also identified a consistent decrease in the mRNA levels of *UCHL5*, a deubiquitinating enzyme that cleaves Lys-48-linked polyubiquitin chains as a final stage prior to subsequent protein degradation in the 26S proteasome (Fig. 5).

## Discussion

Approaches using individual markers to identify CSCs reveal distinct subpopulations of tumor cells and highlight CSC heterogeneity, with properties varying according to cancer type and different regions within the same tumor [6, 27, 47, 48]. Consequently, there is a need to identify general functional properties that distinguish CSCs from the tumor bulk, which is critical for understanding the nature of these cells to advance targeted therapies and enhance patient outcomes [13]. Reduced proteasome activity is a reliable and robust marker of cancer-initiating cells in vitro and in vivo, and targeting these low proteasome activity cells is a therapeutic option [83, 84]. Previous studies have also indicated asymmetric cell division, increased sphere-forming capacity, expression of CSC markers, and a lack of differentiation markers of low proteasome cells [57, 76, 76], supporting their CSC nature. Here, we engineered EmGFP-ODC FaDu HNSCC cells. A key advantage of this model is continual expression of an unstable fluorescent protein targeted specifically to the proteasome, allowing us to couple proteasome activity in live cells with assays of cancer cell phenotypes and their metabolism.

ALDH activity was identified as a CSC marker in various cancers [11, 65]. In particular, ALDH marks a specific sub-population of CSCs with an epithelial-like phenotype [6, 8, 39, 47, 49, 50, 58, 89]. Here, we demonstrated that low proteasome activity in FaDu squamous cell carcinoma cells is positively associated with high ALDH activity and ALDH1A1 protein levels, indicating that proteasome-low CSCs overlap with the ALDH-high epithelial CSC phenotype.

Cancers commonly exhibit the Warburg effect characterized by increased glycolytic activity even in the presence of sufficient oxygen, which enables the production of metabolic intermediates for macromolecular synthesis needed for rapid tumor growth, but requires increased glucose availability [20, 30]. However, because of their relatively slow growth, CSCs may show a more oxidative metabolism [80, 88], and elevated MMP and mitochondrial activity play pivotal roles in shaping stem-like phenotypes [52, 74]. We demonstrated that FaDu CSCs with low proteasome activity exhibit relatively high mitochondrial activity and relatively low glucose transporter levels, indicating that these CSCs show non-Warburg glucose metabolism. These findings are in keeping with the less glycolytic and more oxidative metabolism seen in the ALDH^+^ subtype of CSCs compared to other CSC subtypes [58]. Taken together, the combination of low proteasomal activity with increased mitochondrial activity and/or lower glucose transporter levels serve as functional markers of this specific CSC subtype in HNSCC. The low glucose transporter levels in this cancer cell population has important implications for their selective elimination using glucose metabolism modifying drugs, and for ^18^F-FDG PET detection of CSCs that survive treatment. It will be important in the future to investigate the underlying reasons for the mitochondrial phenotype of proteasome-low CSCs in our study, including real-time metabolic analysis and ultrastructural studies of mitochondrial morphology, with spheroid growth assays, xenograft assays for tumor-initiation, and migration and invasion assays to investigate the cancer-related behavior of these cells further. These additional assays are beyond the scope of the current study but will form the basis for future experiments based on the current findings. Importantly, recent data confirm that cancer cells with high MMP do indeed show the CSC properties of increased anchorage-independent spheroid growth, increased drug reistance, and increased colony formation, along with increased proliferation, invasion/migration, and metastasis in vivo [53]. It is also important to note that our studies did not include cell purification, enabling us to study individual cells under the identical microenvironmental conditions, but precluding analysis of simple cellular characteristics such as cell and mitochondrial morphology, or of tumor initiation and sphere-forming efficiency as surrogate CSC asssays. Further characterization of these cells is required in the future.

While reduced proteasome activity is linked to CSCs phenotypes and heightened therapy resistance [44], the connection between protein synthesis rates and CSC phenotypes remains incomplelety understood. Conversely to initial in vivo studies defining stem cell phenotypes associated with reduction of protein synthesis [5, 10, 34], the majority of transcription and ribosomal protein synthesis occurs within a subset of tumor cells localized to defined niches, defining a common stem cell hierarchy [55]. However, these stem cells are characterized by low translation rates despite the high levels of ribosome biogenesis [36, 67], indicating an altered balance between ribosome content and global protein synthesis. We found that FaDu CSCs identified by low proteasome activity are associated with a lower protein synthesis rate than the majority of tumor cells, meaning that the highly fluorescent cells synthesize less yet have more fluorescent protein than most tumor cells, reinforcing that they possess even less proteasome activity than is measured by the level of EmGFP. Recent data have also suggested that stem cells preferentially employ aggrephagy rather than the proteasome to maintain their strict proteostasis requirements [10]. Our analysis of gene expression profiles of ALDH^+^ CSCs from two ovarian cancer cell lines also indicated that these CSCs display statistically significant differences from the non-CSC populations of these cells, indicating that altered protein turnover is a characteristic feature of these CSCs. Based on these data, further comprehensive resolution of protein synthesis and degradation rates in CSCs warrants further investigation.

Potential therapeutic outcomes emerge from our data. It is clear that cancers show global downregulation of mitochondrial activity and OXPHOS [7], and although glycolysis inhibitors have shown promise for cancer therapy [22, 62], these will be less effective for elimination of CSCs that import less glucose and employ a higher level of mitochondrial activity than the tumor bulk. In contrast, the increased reliance of CSCs on OXPHOS can be targeted by strategies that impair mitochondrial function [70], although this would not affect most cells in the tumor. Thus, the use of both glycolysis and OXPHOS inhibitors is an attractive combination for targeting all cells in the tumor, with evidence for synergistic effects at low doses that do not show toxicicity for non-cancerous cells [70]. In addition, treatment with clinical antgonists of kinases involved in cancer such as BRAF or c-KIT induces OXPHOS-addiction that limits drug efficacy and simultaneously induces sensitivity to OXPHOS inhibition as combination therapy [29, 85]. Similarly, combination of approved antidiabetic agents such as metformin with conventional radio- or chemo-therapy, has shown promise in selectively eliminating CSCs [68, 72], and biguanides are useful for treating experimental and clinical breast cancers [2, 26, 28] NCT01101438; NCT03026517).

Regarding the proteostasis properties of cancer cells and CSCs, inhibition of protein translation disrupts CSC properties, although these are limited clinically due to their toxicity [42, 46]. This is an active area of research, and the translation elongation inhibitor SVC112 acts selectively against HNSCC CSCs, with less toxicity to healthy cells than existing FDA-approved protein synthesis inhibitors [37]. As with glucose metabolism inhibitors, the potential for combining protein synethesis/degradation inhibitors with more conventional therapies has promise. For example, bortezomib combined with other chemotherapeutic agents induces apoptosis in CSCs by impeding the degradation of ubiquitinated proteins, and has been approved for multiple myeloma patients [18, 44]. Unfortunately, bortezomib lacks efficacy in clinical trials for solid tumors, due to resistance from the elevated expression of anti-apoptotic proteins or upregulation of developmental pathways such as Notch [41]. An alternative approach explores the use of second-generation proteasome inhibitors with distinct pharmacologic properties, such as Kyprolis (carfilzomib) or Ninlaro (ixazomib), which have shown clinical activity in bortezomib-resistant cancers [18, 35]. It must also be taken into account that proteostasis in stem cells is not a simple process of synthesis versus degradation [9], but may also involve selective translation capacity together with altered protein degradation through the proteasome and autophagy [5, 71]. In our work, we identified that Hsp90A, the major Hsp90 form, is downregulated in ALDH^+^ CSCs compared to the majority of cancer cells, which are known to rely on high levels of HSP90 activity, including post-translational modifications that promote Hsp90 binding to the co-chaperone STIP1 and inhibit binding to CHIP to increase protein folding and stabilization [56]. We find that the reliance of cancer cells for increased chaperones is selective for the tumor bulk, raising concerns for the application of chaperone inhibitors for cancer treatment [3]. On the other hand, identification of consistent changes in protein homeostasis provides potential markers to identify CSCs, as well as acting as targets for therapeutic application. For example, UCHL5 is a selective deubiquitinase and positive regulator of protein degradation [16, 19] that can be targetd by small molecules to overcome therapy resistant cells as combination therapy [1]. Importantly, UCHL5 promotes glycolysis [86] in keeping with our finding that CSCs show lower *UCHL5* mRNA levels than the tumor bulk together with lower levels of glucose transporter and higher mitochondrial activity. Based on our findings, further research will be required to uncover the involvement of proteostatic processes in cancer cells and the CSC population, and the relationships with glucose metabolism to allow efficient targeting of all cancer cell subsets within a tumor [82].

## Conclusions

This study identified low proteasome activity in combination with reduced glucose transporter levels and elevated MMP as key properties of ALDH^+^ CSCs in the FaDu HNSCC cell line. Examination of gene expression profiing data of other cancer cell lines confirms the relationships between ALDH activity and proteostasis. Our findings also highlight the metabolic heterogeneity and complex relationships between proteasome activity, protein synthesis rates, and glucose metabolism in tumor cells. Combined OXPHOS and selective proteasome inhibition has potential for improving therapies that target the variety of tumor cell subpopulations present in a tumor, and for developing personalized treatments based on specific CSC-related functional properties.

## Methods

### Cell culture and treatments

The squamous carcinoma cell line FaDu (ATCC, VA, USA) was cultured in high glucose Dulbecco's Modified Eagle Medium (DMEM, D5648, Sigma Aldrich), supplemented with penicillin (100 U/ml), streptomycin (100 µg/ml) (Biosera XC-A4122/100; BioTech), 1% sodium pyruvate, and 10% fetal bovine serum (Biosera S-FBS-SA-015). Cell line authentication was performed based on STR analysis (Eurofins Genomics), and regular testing for mycoplasma contamination was conducted by PCR. Cells were maintained in a 95% humidified atmosphere of 5% CO_2_ at 37 °C. Passaging was performed after exposure to EGTA/PBS and trypsin. Cell numbers were determined using a TC20 Automated Cell Counter (BioRad). After seeding at appropriate densities to ensure cells reached a maximum 80% confluency at the end of the experiment, cells were grown for 48 h before treatment for the times and concentrations indicated in the Figure legends.

### Preparation of EmGFP-ODC stable cell line

FaDu cells were cultured to 70–90% confluency. We used a Piggy-Bac transposon vector approach to induce plasmid incoprporation into the genome to produce stable cells. Transfection was performed using equal amounts of PB-EF1a-N-EmGFP-ODC1(418–461)-PURO-GWs plasmid (derived from the construct described in Mistrik et al., [54]) and pRP[Exp]-mCherry-CAG > hyPBase transposase in Opti-MEM medium (Thermo Fisher Scientific) with Lipofectamine P3000 (Thermo Fisher Scientific) following the manufacturer's protocol. EmGFP (Emerald Green fluorescent protein) shows improved photostability and brightness than EGFP, and is targeted for rapid degradation in the proteasome due to introduction of the murine ornithine decarboxylase (ODC) degron sequence at the C-terminus. EmGFP is controlled by the EF1a promoter, providing constitutive expression, and the puromycin resistance cassette is independently driven from a separate constitutive SV40 promoter (Fig. S1). Two days post-transfection, cells were selected through continuous puromycin treatment (2 µg/ml; Invivogen) in complete media. Subsequently, single cells were isolated (BD FACS Aria™ III Cell Sorter) excluding doublets, debris, and dead cells. Single cell clones were expanded by growth in DMEM containing puromycin. Cells were tested for proteasome activity-dependent fluorescence using the proteasome inhibitor MG-132 (Merck) and were maintained under puromycin selection until experimental procedures were performed.

### Live/dead violet viability assay

Cells were stained with LIVE/DEAD™ Fixable Violet Dead Cell Stain Kit (diluted 1:1000, Thermo Fisher Scientific) in PBS, followed by washing, fixation using 3.5% PFA, and permeabilization with 0.1% Triton X-100, or direct staining depending on the experiment. Subsequently, flow cytometry was conducted using FACSVerse™ and FACSuite™ software (version 1.0.5, BD). Cells with impaired plasma membrane integrity (dead cells) were excluded from further analyses.

### Analysis of ALDH enzyme activity and ALDH1A1 levels

The AldeRed Detection Kit (SCR150, Merck) was used for detection of ALDH enzyme activity according to the manufacturer's protocol. The ALDH inhibitor diethylaminobenzaldehyde (DEAB) was used in negative control testing for background fluorescence assessment. FaDu EmGFP-ODC clones exhibited a significant decrease in the number of ALDH^+^ cells in the presence of DEAB (Fig. S3), confirming assay specificity. Alternatively, Alexa Fluor® 647-conjugated ALDH1A1 antibody (ab195255, Abcam) was used for immunofluorescence.

### Measurement of mitochondrial membrane potential (MMP)

MMP was measured according to the manufacturer's protocols using flow cytometry measurement of MitoTracker™ Deep Red FM accumulation (Thermo Fisher Scientific; 100 nM, 30 min at 37 °C). Cells were washed in PBS and analyzed using FACSVerse™/FACSuite™, or FACSAria III™/FACSDiva™ software. For fluorescence microscopy, MitoMark Red (Tocris, 6445, 50 nM, 30 min at room temperature) was utilized, which accumulates in mitochondria dependent on their MMP.

*Measurement of glucose transporter 1 (GLUT-1)* Glucose transporter mRNA levels in FaDu cells were initially assessed using RNA-seq data from the Cancer Cell Line Encyclopedia [23], concentrating on the major glucose transporter in most cancers, GLUT-1 (encoded by *SLC2A1*) [75], and GLUT-3 (*SLC2A3*), previously associated with brain tumour stem cells [14, 21]. These data showed that GLUT-1 mRNA is present at more than 100-fold higher levels than GLUT-3 (106 reads compared to 0.82 reads per kilobase per million mapped reads (RPKM)). Therefore, we measured GLUT-1 levels by flow cytometry in fixed cells using Alexa Fluor® 647-conjugated GLUT-1 antibody (1:500, ab195020, Abcam). Isotype control antibody (CS2985S) was used as a negative control (Fig. S4).

### Measurement of protein synthesis

Protein synthesis was measured by incorporation of puromycin (2 μg/ml, 15 min, 37 °C) into nascent polypeptide chains. Cells were fixed and permeabilized and newly synthesized proteins were detected with monoclonal mouse anti-puromycin (1:2000, Sigma Aldrich, MABE343) and anti-mouse Alexa Fluor® 647 (A32728, Thermo Fisher Scientific; 1:2000). Primary antibody omission and control secondary antibody were used to assess non-specific binding (Fig. S5). In these experiments, selection medium containing puromycin was replaced by medium without puromycin 24 h prior to treatment.

### Immunofluorescence

Cells were seeded on 22 × 22 mm coverslips in 6-well plates. For mitochondrial staining, cells were treated with MitoMark Red (50 nM, 30 min at room temperature), and fixed in 4% formaldehyde for 10 min at room temperature. Cells were permeabilized with 0.1% Triton X-100 and then incubated with blocking buffer (1% BSA in PBS) before staining overnight at 4 °C with Alexa Fluor® 647 ALDH1A1 antibody (1:100, ab195255, Abcam). ProLong™ Glass Antifade Mountant with NucBlue™ Stain (Thermo Fisher P3698) was used for nuclear counterstaining and mounting onto glass slides. Isotype control antibody was used to assess non-specific binding. Samples were examined and photographed using an EVOS FL fluorescence microscope (Thermo Fisher Scientific).

### Statistical analyses

The results of at least three independent experiments are shown as the mean ± SEM. For statistical analysis, cells were gated according to the highest or lowest 5% for each marker, derived from the same denominator, to identify the population containing the putative CSCs (as described in Liu Y et al., [48]). According to the null hypothesis that the two parameters are not related to each other, the double-gated population will contain 0.25% of the total cells (5% of 5%), which is denoted as the “expected” percentage of cells (please note that the manual gating procedure produces slight variations in the actual percentages gated, providing a mean and standard error for the expected values). The "observed" cell percentage represents the experimentally determined percentage of cells within the double-gated population, which is then statistically compared to the expected values using data from the three biological replicates. Please see supplementary information for a more detailed discussion of this approach. Statistical significance was determined by paired two-tailed t-tests. Graphs were created using GraphPad Prism.

### Gene expression profiling

Gene expression profiling data of ALDH-sorted cancer cells were obtained from the public Gene Expression Omnibus (https://www.ncbi.nlm.nih.gov/geo/). We identified two ovarian cancer cell lines, SKOV-3 representing high grade ovarian cystadenocarcinoma and FNAR-C1 representing endometrioid ovarian carcinoma that had been separately purified into ALDH-high and ALDH-low cell populations for expression profiling [69] accession numbers GSE82304 and GSE82112). Gene expression profile values from the two populations of each cell line were retrieved using the GEO2R tool after assigning arrays into either ALDH-high or ALDH-low categories, with individual expression values for the mRNAs of interest obtained using the profile graph and sample values tools. The mean values were calculated for the ALDH-high and ALDH-low populations and statistically compared using 2-tailed paired t-tests for replicate samples (*n* = 3 biological replicates). Graphs show the mean fold-change in mRNA levels between the ALDH-high and ALDH-low populations. Values higher than 1 indicate higher expression in the ALDH-high (CSC) population compared to the ALDH-low (non-CSC) population. The genes selected for analysis represent major HSP70 and HSP90 chaperones (*HSP9A1A, HSPA6*, *HSPA8*, *HSP90AA1*, *HSP90AB1*), stimulatory co-chaperones (*STIP1*, *AHSA1*), inhibitory co-chaperone (*STUB1*) and proteasome subunits *PSMC1*, *PSMD1*, *PSME4*, and *UCHL5*. Note that not all of these genes are measured on the Agilent whole rat genome microarray used for FNAR-C1 analyses (platform GPL14745 in GEO). For these latter analyses, we also downloaded the full annotations of the array platform to allow identification of gene names that correspond to the gene ID number on the array.

## Supplementary Information

Supplementary Material 1.

Supplementary Material 2: S1 Schematic map of the PB-EF1a-N-EmGFP-ODC1(418-461)-PURO-GWs plasmid. S2 Validation of EmGFP-ODC-cells as a measure of proteasome activity. S3 Validation of ALDH enzyme activity assay. S4 Specificity of GLUT1 staining in FaDu EmGFP-ODC clones using isotype control antibody. S5 Assessment of non-specific binding of puromycin antibody in FaDu EmGFP-ODC clones.

## Data Availability

The data produced and analyzed during the current study are included within the article and its additional files. The gene expression profiling data analyzed in this study are deposited in NCBI Gene Expression Omnibus (GEO) and are accessible through accession numbers GSE82304 and GSE82112.

## Abbreviations

AHSA1: AHA1, Activator of HSP90 ATPase Homolog 1
ALDH(1A1): Aldehyde Dehydrogenase(1A1)
CSC: Cancer Stem-like Cell
EmGFP: Emerald Green Fluorescent Protein
GLUT-1: Glucose Transporter 1
HNSCC: Head and Neck Squamous Cell Carcinoma
HSP90AA1: Heat Shock Protein 90 Alpha Family Class A Member 1
HSP90AB1: Heat Shock Protein 90 Alpha Family Class B Member 1
HSPA1A: Heat Shock Protein Family A Member 1A
HSPA6/8: Heat Shock Protein Family A Member 6 and 8
MMP: Mitochondrial membrane potential
ODC: Ornithine Decarboxylase
OXPHOS: Oxidative Phosphorylation
PSMC1: Proteasome 26S Subunit, ATPase 1
PSMD1: Proteasome 26S Subunit, Non-ATPase 1
PSME4: Proteasome Activator Subunit 4
STIP1: Stress-Induced Phosphoprotein 1
STUB1: STIP1 Homology and U-Box Containing Protein 1
UCHL5: Ubiquitin C-Terminal Hydrolase L5
WT: Wild-Type

## References

[1] Amari K, Sasagawa S, Imayoshi N, Toda Y, Hosogi S, Imamura T, Ashihara E. The CDK4/6-UCHL5-BRD4 axis confers resistance to BET inhibitors in MLL-rearranged leukemia cells by suppressing BRD4 protein degradation. Biochem Biophys Res Commun. 2022;15(588):147–53. 10.1016/j.bbrc.2021.12.063 34954522

[2] Appleyard MVCL, Murray KE, Coates PJ, et al. Phenformin as prophylaxis and therapy in breast cancer xenografts. Br J Cancer. 2012;106(6):1117–22. 22361631 10.1038/bjc.2012.56 PMC3304424

[3] Backe SJ, Woodford MR, Ahanin E, Sager RA, Bourboulia D, Mollapour M. Impact of Co-chaperones and Posttranslational Modifications Toward Hsp90 Drug Sensitivity. Subcell Biochem. 2023;101:319–50. 36520312 10.1007/978-3-031-14740-1_11 PMC10077965

[4] Batlle E, Clevers H. Cancer stem cells revisited. Nat Med. 2017;23(10):1124–34. 28985214 10.1038/nm.4409

[5] Blanco S, Bandiera R, Popis M, Hussain S, Lombard P, Aleksic J, et al. Stem cell function and stress response are controlled by protein synthesis. Nature. 2016;534(7607):335–40. 27306184 10.1038/nature18282 PMC5040503

[6] Bocci F, Gearhart-Serna L, Boareto M, Ribeiro M, Ben-Jacob E, Devi GR, et al. Toward understanding cancer stem cell heterogeneity in the tumor microenvironment. PNAS. 2019;116(1):148–57. 30587589 10.1073/pnas.1815345116 PMC6320545

[7] Boykov IN, Montgomery MM, Hagen JT, Aruleba RT, McLaughlin KL, Coalson HS, et al. Pan-tissue mitochondrial phenotyping reveals lower OXPHOS expression and function across cancer types. Sci Rep. 2023;13(1):16742. 37798427 10.1038/s41598-023-43963-5 PMC10556099

[8] Cho KJ, Park EJ, Kim MS, Joo YH. Characterization of FaDu-R, a radioresistant head and neck cancer cell line, and cancer stem cells. Auris Nasus Larynx. 2018;45(3):566–73. 28844650 10.1016/j.anl.2017.07.011

[9] Chua BA, Van Der Werf I, Jamieson C, Signer RAJ. Post-Transcriptional Regulation of Homeostatic, Stressed, and Malignant Stem Cells. Cell Stem Cell. 2020;26(2):138–59. 32032524 10.1016/j.stem.2020.01.005 PMC7158223

[10] Chua BA, Lennan CJ, Sunshine MJ, Dreifke D, Chawla A, Bennett EJ, et al. Hematopoietic stem cells preferentially traffic misfolded proteins to aggresomes and depend on aggrephagy to maintain protein homeostasis. Cell Stem Cell. 2023;30(4):460-472.e6. 36948186 10.1016/j.stem.2023.02.010 PMC10164413

[11] Clark DW, Palle K. Aldehyde dehydrogenases in cancer stem cells: potential as therapeutic targets. Ann Transl Med. 2016;4(24):518–518. 28149880 10.21037/atm.2016.11.82 PMC5233526

[12] Clarke MF. Clinical and Therapeutic Implications of Cancer Stem Cells. N Engl J Med. 2019;380(23):2237–45. 31167052 10.1056/NEJMra1804280

[13] Clevers H, Watt FM. Defining Adult Stem Cells by Function, not by Phenotype. Annu Rev Biochem. 2018;87(1):1015–27. 29494240 10.1146/annurev-biochem-062917-012341

[14] Cosset É, Ilmjärv S, Dutoit V, Elliott K, von Schalscha T, Camargo MF, Reiss A, Moroishi T, Seguin L, Gomez G, Moo JS, Preynat-Seauve O, Krause KH, Chneiweiss H, Sarkaria JN, Guan KL, Dietrich PY, Weis SM, Mischel PS, Cheresh DA. Glut3 Addiction Is a Druggable Vulnerability for a Molecularly Defined Subpopulation of Glioblastoma. Cancer Cell. 2017;32(6):856-868.e5. 29198914 10.1016/j.ccell.2017.10.016 PMC5730343

[15] Della Donna L, Lagadec C, Pajonk F. Radioresistance of prostate cancer cells with low proteasome activity. Prostate. 2012;72(8):868–74. 21932424 10.1002/pros.21489 PMC3396561

[16] Deol KK, Crowe SO, Du J, Bisbee HA, Guenette RG, Strieter ER. Proteasome-Bound UCH37/UCHL5 Debranches Ubiquitin Chains to Promote Degradation. Mol Cell. 2020;80(5):796-809.e9. 33156996 10.1016/j.molcel.2020.10.017 PMC7718437

[17] dos Santos RV, da Silva LM. A possible explanation for the variable frequencies of cancer stem cells in tumors. PLoS ONE. 2013;8(8):e69131. 23950884 10.1371/journal.pone.0069131 PMC3737222

[18] Dou Q, Zonder J. Overview of proteasome inhibitor-based anti-cancer therapies: perspective on bortezomib and second generation proteasome inhibitors versus future generation inhibitors of ubiquitin-proteasome system. CCDT. 2014;14(6):517–36. 10.2174/1568009614666140804154511 PMC4279864 25092212

[19] Du J, Babik S, Li Y, Deol KK, Eyles SJ, Fejzo J, Tonelli M, Strieter E. A cryptic K48 ubiquitin chain binding site on UCH37 is required for its role in proteasomal degradation. Elife. 2022;22(11):e76100. 10.7554/eLife.76100 PMC9033301 35451368

[20] Fendt SM. 100 years of the Warburg effect: A cancer metabolism endeavor. Cell. 2024;187(15):3824–8. 39059359 10.1016/j.cell.2024.06.026

[21] Flavahan WA, Wu Q, Hitomi M, Rahim N, Kim Y, Sloan AE, Weil RJ, Nakano I, Sarkaria JN, Stringer BW, Day BW, Li M, Lathia JD, Rich JN, Hjelmeland AB. Brain tumor initiating cells adapt to restricted nutrition through preferential glucose uptake. Nat Neurosci. 2013;16(10):1373–82. 23995067 10.1038/nn.3510 PMC3930177

[22] Ganapathy-Kanniappan S, Geschwind JFH. Tumor glycolysis as a target for cancer therapy: progress and prospects. Mol Cancer. 2013;12(1):152. 24298908 10.1186/1476-4598-12-152 PMC4223729

[23] Ghandi M, Huang FW, Jané-Valbuena J, Kryukov GV, Lo CC, McDonald ER 3rd, Barretina J, Gelfand ET, Bielski CM, Li H, Hu K, Andreev-Drakhlin AY, Kim J, Hess JM, Haas BJ, Aguet F, Weir BA, Rothberg MV, Paolella BR, Lawrence MS, Akbani R, Lu Y, Tiv HL, Gokhale PC, de Weck A, Mansour AA, Oh C, Shih J, Hadi K, Rosen Y, Bistline J, Venkatesan K, Reddy A, Sonkin D, Liu M, Lehar J, Korn JM, Porter DA, Jones MD, Golji J, Caponigro G, Taylor JE, Dunning CM, Creech AL, Warren AC, McFarland JM, Zamanighomi M, Kauffmann A, Stransky N, Imielinski M, Maruvka YE, Cherniack AD, Tsherniak A, Vazquez F, Jaffe JD, Lane AA, Weinstock DM, Johannessen CM, Morrissey MP, Stegmeier F, Schlegel R, Hahn WC, Getz G, Mills GB, Boehm JS, Golub TR, Garraway LA, Sellers WR. Next-generation characterization of the Cancer Cell Line Encyclopedia. Nature. 2019;569(7757):503–8. 31068700 10.1038/s41586-019-1186-3 PMC6697103

[24] Gil Vazquez E, Nasreddin N, Valbuena GN, Mulholland EJ, Belnoue-Davis HL, Eggington HR, et al. Dynamic and adaptive cancer stem cell population admixture in colorectal neoplasia. Cell Stem Cell. 2022;29(8):1213-1228.e8. 35931031 10.1016/j.stem.2022.07.008 PMC9592560

[25] Ginestier C, Hur MH, Charafe-Jauffret E, Monville F, Dutcher J, Brown M, et al. ALDH1 Is a Marker of Normal and Malignant Human Mammary Stem Cells and a Predictor of Poor Clinical Outcome. Cell Stem Cell. 2007;1(5):555–67. 18371393 10.1016/j.stem.2007.08.014 PMC2423808

[26] Goodwin PJ, Chen BE, Gelmon KA, Whelan TJ, Ennis M, Lemieux J, et al. Effect of Metformin vs placebo on invasive disease-free survival in patients with breast cancer: the MA.32 randomized clinical trial. JAMA. 2022;327(20):1963. 35608580 10.1001/jama.2022.6147 PMC9131745

[27] Gupta PB, Pastushenko I, Skibinski A, Blanpain C, Kuperwasser C. Phenotypic Plasticity: Driver of Cancer Initiation, Progression, and Therapy Resistance. Cell Stem Cell. 2019;24(1):65–78. 30554963 10.1016/j.stem.2018.11.011 PMC7297507

[28] Hadad SM, Coates P, Jordan LB, Dowling RJO, Chang MC, Done SJ, et al. Evidence for biological effects of metformin in operable breast cancer: biomarker analysis in a pre-operative window of opportunity randomized trial. Breast Cancer Res Treat. 2015;150(1):149–55. 25682077 10.1007/s10549-015-3307-5

[29] Haq R, Shoag J, Andreu-Perez P, Yokoyama S, Edelman H, Rowe GC, et al. Oncogenic BRAF regulates oxidative metabolism via PGC1α and MITF. Cancer Cell. 2013;23(3):302–15. 23477830 10.1016/j.ccr.2013.02.003 PMC3635826

[30] Hardie DG. 100 years of the Warburg effect: a historical perspective. Endocr-Relat Cancer. 2022;29(12):T1-13. 36094878 10.1530/ERC-22-0173

[31] Harper LJ, Piper K, Common J, Fortune F, Mackenzie IC. Stem cell patterns in cell lines derived from head and neck squamous cell carcinoma. J Oral Pathol Med. 2007;36(10):594–603. 17944752 10.1111/j.1600-0714.2007.00617.x

[32] Hay N. Reprogramming glucose metabolism in cancer: can it be exploited for cancer therapy? Nat Rev Cancer. 2016;16(10):635–49. 27634447 10.1038/nrc.2016.77 PMC5516800

[33] Hayashi K, Tamari K, Ishii H, Konno M, Nishida N, Kawamoto K, et al. Visualization and characterization of cancer stem-like cells in cervical cancer. Int J Oncol. 2014;45(6):2468–74. 25269542 10.3892/ijo.2014.2670

[34] Hidalgo San Jose L, Signer RAJ. Cell-type-specific quantification of protein synthesis in vivo. Nat Protoc. 2019;14(2):441–60. 30610239 10.1038/s41596-018-0100-z PMC8105873

[35] Ito S. Proteasome Inhibitors for the Treatment of Multiple Myeloma. Cancers. 2020;12(2):265. 31979059 10.3390/cancers12020265 PMC7072336

[36] Jarzebowski L, Le Bouteiller M, Coqueran S, Raveux A, Vandormael-Pournin S, David A, et al. Mouse adult hematopoietic stem cells actively synthesize ribosomal RNA. RNA. 2018;24(12):1803–12. 30242063 10.1261/rna.067843.118 PMC6239186

[37] Keysar SB, Gomes N, Miller B, Jackson BC, Le PN, Morton JJ, et al. Inhibiting translation elongation with SVC112 suppresses cancer stem cells and inhibits growth in head and neck squamous carcinoma. Cancer Res. 2020;80(5):1183–98. 31911553 10.1158/0008-5472.CAN-19-3232 PMC7056512

[38] Kim J, Villadsen R, Sørlie T, Fogh L, Grønlund SZ, Fridriksdottir AJ, et al. Tumor initiating but differentiated luminal-like breast cancer cells are highly invasive in the absence of basal-like activity. PNAS. 2012;109(16):6124–9. 22454501 10.1073/pnas.1203203109 PMC3341000

[39] Kurth I, Hein L, Mäbert K, Peitzsch C, Koi L, Cojoc M, Kunz-Schughart L, Baumann M, Dubrovska A. Cancer stem cell related markers of radioresistance in head and neck squamous cell carcinoma. Oncotarget. 2015;6(33):34494–509. 26460734 10.18632/oncotarget.5417 PMC4741468

[40] Lagadec C, Vlashi E, Bhuta S, Lai C, Mischel P, Werner M, et al. Tumor cells with low proteasome subunit expression predict overall survival in head and neck cancer patients. BMC Cancer. 2014;14(1): 152. 24593279 10.1186/1471-2407-14-152 PMC3975871

[41] Lagadec C, Vlashi E, Frohnen P, Alhiyari Y, Chan M, Pajonk F. The RNA-Binding Protein Musashi-1 Regulates Proteasome Subunit Expression in Breast Cancer- and Glioma-Initiating Cells. Stem Cells. 2014;32(1):135–44. 24022895 10.1002/stem.1537 PMC3968686

[42] Laham-Karam N, Pinto GP, Poso A, Kokkonen P. Transcription and Translation Inhibitors in Cancer Treatment. Front Chem. 2020;8:276. 32373584 10.3389/fchem.2020.00276 PMC7186406

[43] Lee JH, Jung KH, Park JW, Moon SH, Cho YS, Lee KH. Targeting poor proteasomal function with radioiodine eliminates CT26 colon cancer stem cells resistant to bortezomib therapy. Sci Rep. 2020;10(1):14308. 32868872 10.1038/s41598-020-71366-3 PMC7459321

[44] Lenos KJ, Vermeulen L. Cancer stem cells don’t waste their time cleaning—low proteasome activity, a marker for cancer stem cell function. Ann Transl Med. 2016;4(24):519–519. 28149881 10.21037/atm.2016.11.81 PMC5233536

[45] Li H, Chen X, Calhoun-Davis T, Claypool K, Tang DG. PC3 human prostate carcinoma cell holoclones contain self-renewing tumor-initiating cells. Cancer Res. 2008;68(6):1820–5. 18339862 10.1158/0008-5472.CAN-07-5878

[46] Lindqvist LM, Vikström I, Chambers JM, McArthur K, Ann Anderson M, Henley KJ, et al. Translation inhibitors induce cell death by multiple mechanisms and Mcl-1 reduction is only a minor contributor. Cell Death Dis. 2012;3(10):e409–e409. 23059828 10.1038/cddis.2012.149 PMC3481137

[47] Liu S, Cong Y, Wang D, Sun Y, Deng L, Liu Y, et al. Breast Cancer Stem Cells Transition between Epithelial and Mesenchymal States Reflective of their Normal Counterparts. Stem Cell Rep. 2014;2(1):78–91. 10.1016/j.stemcr.2013.11.009 PMC3916760 24511467

[48] Liu Y, Nenutil R, Appleyard MV, Murray K, Boylan M, Thompson AM, et al. Lack of correlation of stem cell markers in breast cancer stem cells. Br J Cancer. 2014;110(8):2063–71. 24577057 10.1038/bjc.2014.105 PMC3992489

[49] Liu Y, Nekulova M, Nenutil R, Horakova I, Appleyard MV, Murray K, et al. ∆Np63/p40 correlates with the location and phenotype of basal/mesenchymal cancer stem-like cells in human ER ^+^ and HER2 ^+^ breast cancers. J Pathol Clin Res. 2020;6(1):83–93. 31591823 10.1002/cjp2.149 PMC6966710

[50] Mah V, Elshimali Y, Chu A, Moatamed NA, Uzzell JP, Tsui J, et al. ALDH1 expression predicts progression of premalignant lesions to cancer in Type I endometrial carcinomas. Sci Rep. 2021;11(1):11949. 34099751 10.1038/s41598-021-90570-3 PMC8184965

[51] Maitland NJ, Collins AT. Prostate Cancer Stem Cells: A New Target for Therapy. J Clin Oncol. 2008;26(17):2862–70. 18539965 10.1200/JCO.2007.15.1472

[52] Mansell E, Sigurdsson V, Deltcheva E, Brown J, James C, Miharada K, et al. Mitochondrial Potentiation Ameliorates Age-Related Heterogeneity in Hematopoietic Stem Cell Function. Cell Stem Cell. 2021;28(2):241-256.e6. 33086034 10.1016/j.stem.2020.09.018

[53] Mauro-Lizcano M, Di Pisa F, Larrea Murillo L, Sugden CJ, Sotgia F, Lisanti MP. High mitochondrial DNA content is a key determinant of stemness, proliferation, cell migration, and cancer metastasis in vivo. Cell Death Dis. 2024;15(10):745. 39394145 10.1038/s41419-024-07103-9 PMC11470112

[54] Mistrik M, Skrott Z, Muller P, Panacek A, Hochvaldova L, Chroma K, Buchtova T, Vandova V, Kvitek L, Bartek J. Microthermal-induced subcellular-targeted protein damage in cells on plasmonic nanosilver-modified surfaces evokes a two-phase HSP-p97/VCP response. Nat Commun. 2021;12(1):713. 33514738 10.1038/s41467-021-20989-9 PMC7846584

[55] Morral C, Stanisavljevic J, Hernando-Momblona X, Mereu E, Álvarez-Varela A, Cortina C, et al. Zonation of Ribosomal DNA Transcription Defines a Stem Cell Hierarchy in Colorectal Cancer. Cell Stem Cell. 2020;26(6):845-861.e12. 32396863 10.1016/j.stem.2020.04.012 PMC9006079

[56] Muller P, Ruckova E, Halada P, Coates PJ, Hrstka R, Lane DP, Vojtesek B. C-terminal phosphorylation of Hsp70 and Hsp90 regulates alternate binding to co-chaperones CHIP and HOP to determine cellular protein folding/degradation balances. Oncogene. 2013;32(25):3101–10. 22824801 10.1038/onc.2012.314

[57] Munakata K, Uemura M, Tanaka S, Kawai K, Kitahara T, Miyo M, et al. Cancer stem-like properties in colorectal cancer cells with low proteasome activity. Clin Cancer Res. 2016;22(21):5277–86. 27166395 10.1158/1078-0432.CCR-15-1945

[58] Nimmakayala RK, Leon F, Rachagani S, Rauth S, Nallasamy P, Marimuthu S, et al. Metabolic programming of distinct cancer stem cells promotes metastasis of pancreatic ductal adenocarcinoma. Oncogene. 2021;40(1):215–31. 33110235 10.1038/s41388-020-01518-2 PMC10041665

[59] Oshimori N, Guo Y, Taniguchi S. An emerging role for cellular crosstalk in the cancer stem cell niche. J Pathol. 2021;254(4):384–94. 33634866 10.1002/path.5655 PMC9575701

[60] Pan J, Zhang Q, Wang Y, You M. 26S Proteasome Activity Is Down-Regulated in Lung Cancer Stem-Like Cells Propagated In Vitro. Wang X, editor. PLoS ONE. 2010;5(10):e13298. 20949018 10.1371/journal.pone.0013298 PMC2952619

[61] Pavlova NN, Zhu J, Thompson CB. The hallmarks of cancer metabolism: Still emerging. Cell Metab. 2022;34(3):355–77. 35123658 10.1016/j.cmet.2022.01.007 PMC8891094

[62] Pelicano H, Martin DS, Xu RH, Huang P. Glycolysis inhibition for anticancer treatment. Oncogene. 2006;25(34):4633–46. 16892078 10.1038/sj.onc.1209597

[63] Pendergrass W, Wolf N, Poot M. Efficacy of MitoTracker Green™ and CMXrosamine to measure changes in mitochondrial membrane potentials in living cells and tissues. Cytom A. 2004;61A(2):162–9. 10.1002/cyto.a.20033 15382028

[64] Pérez-González A, Bévant K, Blanpain C. Cancer cell plasticity during tumor progression, metastasis and response to therapy. Nat Cancer. 2023;4(8):1063–82. 37537300 10.1038/s43018-023-00595-y PMC7615147

[65] Poturnajova M, Kozovska Z, Matuskova M. Aldehyde dehydrogenase 1A1 and 1A3 isoforms – mechanism of activation and regulation in cancer. Cell Signal. 2021;87: 110120. 34428540 10.1016/j.cellsig.2021.110120 PMC8505796

[66] Prasetyanti PR, Medema JP. Intra-tumor heterogeneity from a cancer stem cell perspective. Mol Cancer. 2017;16(1): 41. 28209166 10.1186/s12943-017-0600-4 PMC5314464

[67] Saba JA, Liakath-Ali K, Green R, Watt FM. Translational control of stem cell function. Nat Rev Mol Cell Biol. 2021;22(10):671–90. 34272502 10.1038/s41580-021-00386-2

[68] Sancho P, Barneda D, Heeschen C. Hallmarks of cancer stem cell metabolism. Br J Cancer. 2016;114(12):1305–12. 27219018 10.1038/bjc.2016.152 PMC4984474

[69] Sharrow AC, Perkins B, Collector MI, Yu W, Simons BW, Jones RJ. Characterization of aldehyde dehydrogenase 1 high ovarian cancer cells: towards targeted stem cell therapy. Gynecol Oncol. 2016;142(2):341–8. 27017984 10.1016/j.ygyno.2016.03.022 PMC5843190

[70] Sica V, Bravo-San Pedro JM, Stoll G, Kroemer G. Oxidative phosphorylation as a potential therapeutic target for cancer therapy. Int J Cancer. 2020;146(1):10–7. 31396957 10.1002/ijc.32616

[71] Signer RAJ, Qi L, Zhao Z, Thompson D, Sigova AA, Fan ZP, et al. The rate of protein synthesis in hematopoietic stem cells is limited partly by 4E-BPs. Genes Dev. 2016;30(15):1698–703. 27492367 10.1101/gad.282756.116 PMC5002975

[72] Snyder V, Reed-Newman TC, Arnold L, Thomas SM, Anant S. Cancer Stem Cell Metabolism and Potential Therapeutic Targets. Front Oncol. 2018;8:203. 29922594 10.3389/fonc.2018.00203 PMC5996058

[73] Storms RW, Trujillo AP, Springer JB, Shah L, Colvin OM, Ludeman SM, et al. Isolation of primitive human hematopoietic progenitors on the basis of aldehyde dehydrogenase activity. Proc Natl Acad Sci U S A. 1999;96(16):9118–23. 10430905 10.1073/pnas.96.16.9118 PMC17742

[74] Sukumar M, Liu J, Mehta GU, Patel SJ, Roychoudhuri R, Crompton JG, et al. Mitochondrial Membrane Potential Identifies Cells with Enhanced Stemness for Cellular Therapy. Cell Metab. 2016;23(1):63–76. 26674251 10.1016/j.cmet.2015.11.002 PMC4747432

[75] Szablewski L. Expression of glucose transporters in cancers. BBA - Reviews on Cancer. 2013;1835(2):164–9. 23266512 10.1016/j.bbcan.2012.12.004

[76] Tamari K, Hayashi K, Ishii H, Kano Y, Konno M, Kawamoto K, et al. Identification of chemoradiation-resistant osteosarcoma stem cells using an imaging system for proteasome activity. Int J Oncol. 2014;45(6):2349–54. 25269626 10.3892/ijo.2014.2671

[77] Tian H, Biehs B, Warming S, Leong KG, Rangell L, Klein OD, et al. A reserve stem cell population in small intestine renders Lgr5-positive cells dispensable. Nature. 2011;478(7368):255–9. 21927002 10.1038/nature10408 PMC4251967

[78] Tsvetkov P, Adler J, Myers N, Biran A, Reuven N, Shaul Y. Oncogenic addiction to high 26S proteasome level. Cell Death Dis. 2018;9(7):773. 29991718 10.1038/s41419-018-0806-4 PMC6039477

[79] Vassalli G. Aldehyde Dehydrogenases: Not Just Markers, but Functional Regulators of Stem Cells. Stem Cells Int. 2019;2019:1–15. 10.1155/2019/3904645 PMC6348814 30733805

[80] Viale A, Draetta GF. Sugar? No thank you, just a deep breath of oxygen for cancer stem cells. Cell Metab. 2015;22(4):543–5. 26445511 10.1016/j.cmet.2015.09.020

[81] Visvader JE, Lindeman GJ. Cancer stem cells in solid tumours: accumulating evidence and unresolved questions. Nat Rev Cancer. 2008;8(10):755–68. 18784658 10.1038/nrc2499

[82] Visvader JE, Lindeman GJ. Cancer stem cells: current status and evolving complexities. Cell Stem Cell. 2012;10(6):717–28. 22704512 10.1016/j.stem.2012.05.007

[83] Vlashi E, Kim K, Lagadec C, Donna LD, McDonald JT, Eghbali M, et al. In vivo imaging, tracking, and targeting of cancer stem cells. J Natl Cancer Inst. 2009;101(5):350–9. 19244169 10.1093/jnci/djn509 PMC2727141

[84] Vlashi E, Lagadec C, Chan M, Frohnen P, McDonald AJ, Pajonk F. Targeted elimination of breast cancer cells with low proteasome activity is sufficient for tumor regression. Breast Cancer Res Treat. 2013;141(2):197–203. 24013708 10.1007/s10549-013-2688-6 PMC3814133

[85] Vitiello GA, Medina BD, Zeng S, Bowler TG, Zhang JQ, Loo JK, et al. Mitochondrial Inhibition Augments the Efficacy of Imatinib by Resetting the Metabolic Phenotype of Gastrointestinal Stromal Tumor. Clin Cancer Res. 2018;24(4):972–84. 29246941 10.1158/1078-0432.CCR-17-2697 PMC5815929

[86] Wan B, Cheng M, He T, Zhang L. UCHL5 promotes hepatocellular carcinoma progression by promoting glycolysis through activating Wnt/β-catenin pathway. BMC Cancer. 2024;24(1):618. 38773433 10.1186/s12885-023-11317-z PMC11110341

[87] Yadav UP, Singh T, Kumar P, Sharma P, Kaur H, Sharma S, et al. Metabolic Adaptations in Cancer Stem Cells. Front Oncol. 2020;10:1010. 32670883 10.3389/fonc.2020.01010 PMC7330710

[88] Yasuda T, Ishimoto T, Baba H. Conflicting metabolic alterations in cancer stem cells and regulation by the stromal niche. Regener Ther. 2021;17:8–12. 10.1016/j.reth.2021.01.005 PMC7851492 33598509

[89] Yeo SK, Zhu X, Okamoto T, Hao M, Wang C, Lu P, et al. Single-cell RNA-sequencing reveals distinct patterns of cell state heterogeneity in mouse models of breast cancer. eLife. 2020;9:e58810. 32840210 10.7554/eLife.58810 PMC7447441

