# Supplementary material for: Heterogeneous protein dynamics links to mitochondrial activity, glucose transporter, and ALDH cancer stem cell properties
Source: BMC Cancer. 2025 Jul 1;25:1085. doi: 10.1186/s12885-025-14460-x (PMC12210997; doi:10.1186/s12885-025-14460-x)
Supplement: Supplementary file 1 — Supplementary Material 1. [file 12885_2025_14460_MOESM1_ESM.docx]

**Supplementary information for**

**Heterogeneous Protein Dynamics Links to Mitochondrial Activity, Glucose Transporter, and ALDH Cancer Stem Cell Properties**

Martin Krkoška, Zuzana Tylichová, Pavlína Zatloukalová, Petr Müller, Bořivoj Vojtěšek, and Philip John Coates

Supplementary Methods

*Choice of 5% cut-off for analysis of phenotype*

The use of a 5% cut-off for each parameter is based on multiple observations that CSCs exist as a small subpopulation of cells (generally estimated as approximately 2% to 10% but ranging from less than 0.1% to as much as 40%, depending on the assay system and cancer type (dos Santos and da Silva, 2013; Harper LJ et al., 2007; Ginestier C et al., 2007; Visvader JE and Lindeman GJ, 2008)), and the selection of 5% as threshold has been used previously to good effect (Liu Y et al., 2014). Without precisely defining the exact population of CSCs, this cut-off provides significant enrichment of the CSC population, whether CSCs actually represent <5% or >5% of the total. For example, if 10% of the total cells are in fact CSCs, all cells in the 5% analysed population will be CSCs, and the remaining 95% of cells will contain only 5% CSCs, representing 100% of the analysed population being CSCs compared to 5.5% of the “non-CSC” population representing true CSCs, a 19-fold enrichment. Alternatively, if the CSC population is 2%, then 40% of the analysed CSC population are CSCs and 0% of the CSCs are present in the other, non-CSC population. These levels of enrichment are sufficient to analyse whether or not two parameters are related or unrelated to each other using our statistical approach. Thus, we chose to use a rational approximation for all analyses rather than using a range of different cut-offs for each population to avoid the problem of multiple analyses, where one or other assessment may produce a p value < 0.05 by chance due to multiple testing.

*Statistical analyses.*

Our analyses employ flow cytometric analysis of two variables, derived from the same denominator, and we use a 5% cut-off value to enrich for the proposed CSC phenotype. Using this approach, 0.25% of the cells will be present within the double gated region (5% x 5%) if the two parameters are unrelated, representing the null hypothesis. This “expected” value is then compared to the value “observed” in our experimental results to investigate whether the null hypothesis that the parameters are unrelated is true. However, the 5% gates for each parameter are drawn manually for each replicate in each experimental set up. Therefore, the actual percentage of cells included in the analysed gate is slightly variable, meaning that the “expected” values are also slightly variable depending on the precise placing of the two gates, and this must also be taken into account in the statistical analyses. For example, if the first (vertical) gate is actually drawn at 4.9% and the second (horizontal) gate at 4.9%, the expected percentage is 4.9% x 4.9% = 0.24% for that replicate. For the second replicate, the manual gates may be 5.1 and 5.1, giving 0.26%, and the third set of gates could be 4.85 and 4.9 (=0.23765%). Therefore, the expected values for these three replicates are used as paired values with the corresponding observed values for each replicate to produce a p value that tests deviation from the null hypothesis. Within this, both “expected” and “observed” values are shown as mean values ± standard error of the mean in the graphs.

**References**

dos Santos RV, da Silva LM. A possible explanation for the variable frequencies of cancer stem cells in tumors. PLoS One. 2013 Aug 7;8(8):e69131. doi: 10.1371/journal.pone.0069131

Ginestier C, Hur MH, Charafe-Jauffret E, Monville F, Dutcher J, Brown M, et al. ALDH1 is a marker of normal and malignant human mammary stem cells and a predictor of poor clinical outcome. Cell Stem Cell. 2007 Nov; 1(5):555-67.

Harper LJ, Piper K, Common J, Fortune F, Mackenzie IC. Stem cell patterns in cell lines derived from head and neck squamous cell carcinoma. J Oral Pathol Med. 2007 Nov; 36(10):594-603.

Liu Y, Nenutil R, Appleyard MV, Murray K, Boylan M, Thompson AM, et al. Lack of correlation of stem cell markers in breast cancer stem cells. Br J Cancer. 2014; 110(8):2063–71.

Visvader JE, Lindeman GJ. Cancer stem cells in solid tumours: accumulating evidence and unresolved questions. Nat Rev Cancer. 2008 Oct; 8(10):755-68.
