# Supplementary material for: Heterogeneous protein dynamics links to mitochondrial activity, glucose transporter, and ALDH cancer stem cell properties
Source: BMC Cancer. 2025 Jul 1;25:1085. doi: 10.1186/s12885-025-14460-x (PMC12210997; doi:10.1186/s12885-025-14460-x)
Supplement: Supplementary file 2 — Supplementary Material 2: S1 Schematic map of the PB-EF1a-N-EmGFP-ODC1(418-461)-PURO-GWs plasmid. S2 Validation of EmGFP-ODC-cells as a measure of proteasome activity. S3 Validation of ALDH enzyme activity assay. S4 Specificity of GLUT1 staining in FaDu EmGFP-ODC clones using isotype control antibody. S5 Assessment of non-specific binding of puromycin antibody in FaDu EmGFP-ODC clones. [file 12885_2025_14460_MOESM2_ESM.pdf]

Supplementary Figures

Heterogeneous Protein Dynamics Links to Mitochondrial Activity, Glucose Transporter, and ALDH Cancer Stem Cell Properties

Martin Krkoška[1*], Zuzana Tylichová[1], Pavlína Zatloukalová[1], Petr Müller[1], Bořivoj Vojtěšek[1] and Philip John Coates[1*]

[1] *Research Centre for Applied Molecular Oncology, Masaryk Memorial Cancer Institute, Brno, Czech Republic*

[*] Correspondence to: Martin Krkoška or Philip John Coates, Research Centre for Applied Molecular Oncology, Masaryk Memorial Cancer Institute, Žlutý kopec 7, 656 53 Brno, Czech Republic; e-mail: martin.krkoska@mou.cz or philip.coates@mou.cz; phone: +420 543 133 307

Fig. S1

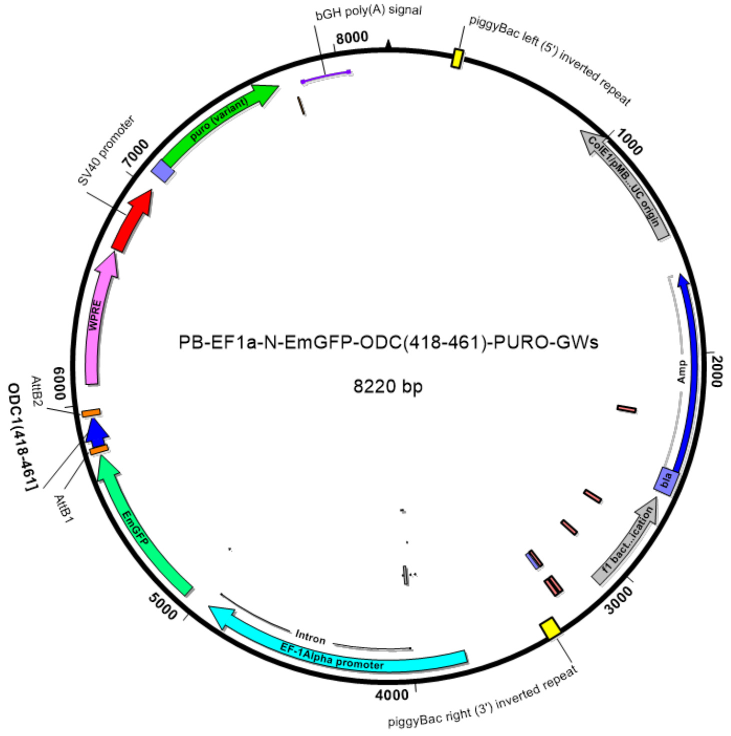

Fig. S1 Schematic map of the PB-EF1a-N-EmGFP-ODC1(418-461)-PURO-GWs plasmid. Schematic map of the PB-EF1a-N-EmGFP-ODC1(418-461)-PURO-GWs plasmid used in this study, showing separate promoters (EF1α for EmGFP-ODC and SV40 for puromycin resistance gene) that drive independent expression of the two genes.

Fig. S2

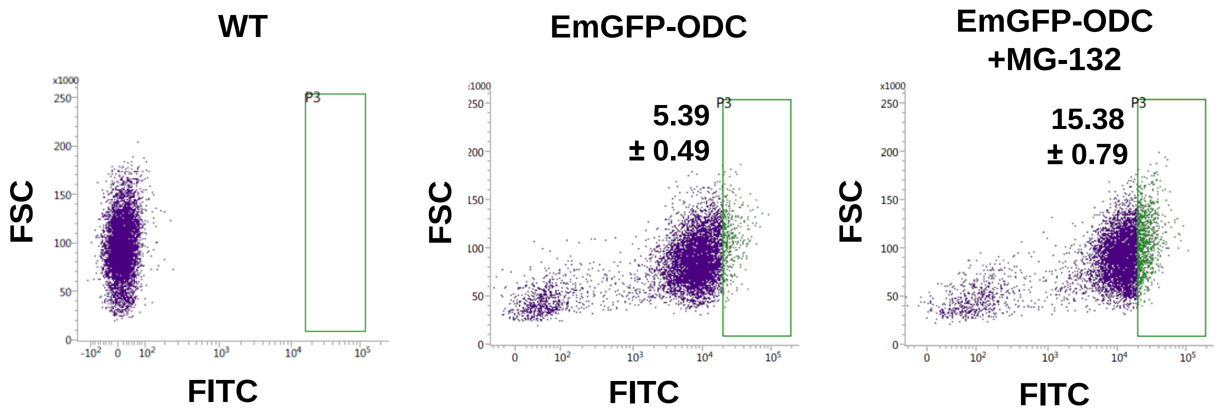

Fig. S2 Validation of EmGFP-ODC-cells as a measure of proteasome activity. Inhibition of the proteasome activity increased the number of EmGFP-ODC-positive cells within the putative CSC-like population. An increase in the number of FITC-positive cells (green) is demonstrated for FaDu EmGFP-ODC clones following MG-132 pretreatment (5 μM, 4 h), in comparison to untreated (DMSO) and untransfected WT cells. These results are representative of a minimum of three independent experiments.

Fig. S3

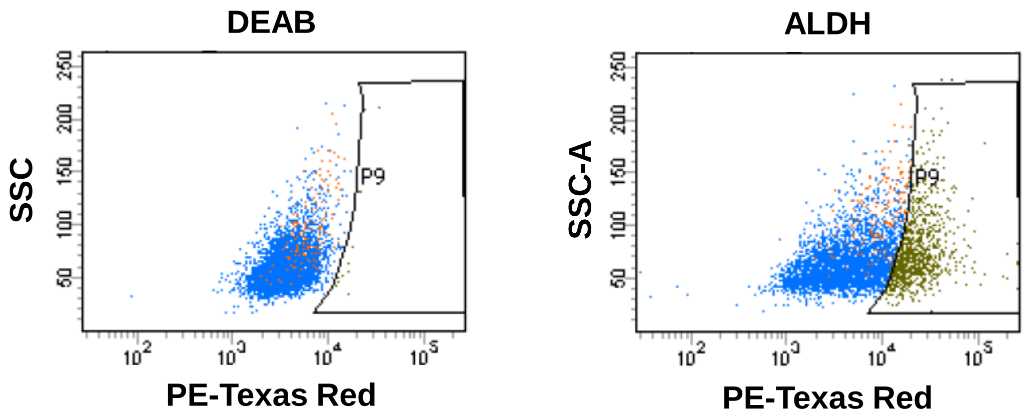

Fig. S3 Validation of ALDH enzyme activity assay. The number of ALDH positive cells (olive) measured in the PE-Texas Red channel on the x axis is plotted against side scatter on the y axis. Data are shown for FaDu EmGFP-ODC clones compared to their respective DEAB controls used for background fluorescence assessment. These results are representative of a minimum of three independent experiments.

Fig. S4

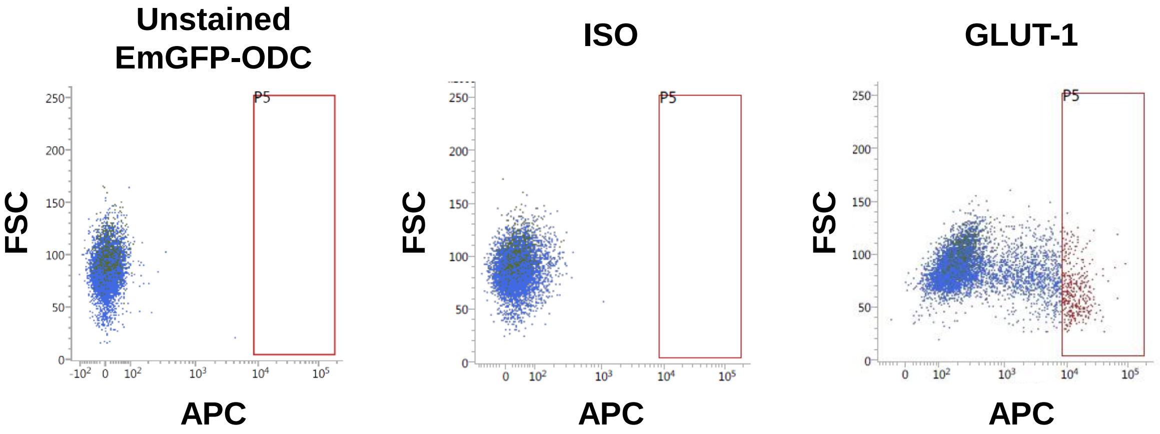

Fig. S4 Specificity of GLUT1 staining in FaDu EmGFP-ODC clones using isotype control antibody. Isotype control antibody (CS2985S) was used in FaDu EmGFP-ODC clones to ensure the specificity of staining with the recombinant Alexa Fluor® 647-GLUT1 antibody (ab195020, 1:500), measured in the APC channel. This was compared to FaDu EmGFP-ODC clones stained with Alexa Fluor® 647-GLUT1 antibody and unstained EmGFP-ODC cells. Gate P5 (red) represents the top 5% of GLUT-1 positive cells.

Fig. S5

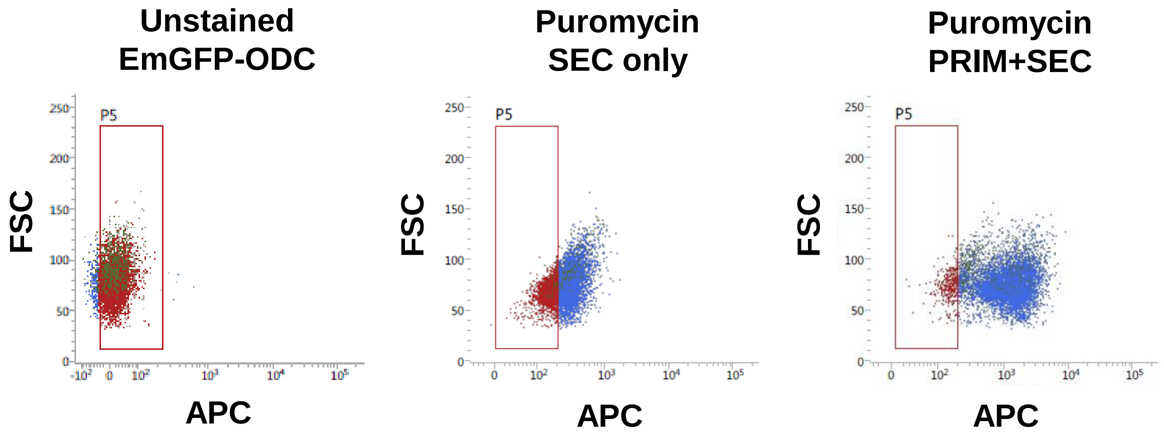

Fig. S5 Assessment of non-specific binding of Puromycin antibody in FaDu EmGFP-ODC clones. To evaluate non-specific binding of the Puromycin antibody (MABE343, 1:2000), primary and secondary antibody omission (Unstained) and a control with only the secondary antibody (Puromycin SEC only) were used in FaDu EmGFP-ODC clones. These conditions were compared to fully stained clones (PRIM+SEC). Gate P5 (red) represents the lowest 5% of puromycin-positive cells in the fully stained sample (PRIM+SEC). Results are representative of at least three independent experiments.
